# Editorial

**DOI:** 10.2478/intox-2013-0006

**Published:** 2013-03

**Authors:** Michal Dubovický

**Affiliations:** Institute of Experimental Pharmacology & Toxicology, Slovak Academy of Sciences, Bratislava, Slovak Republic

*This issue of Interdisciplinary Toxicology is dedicated to the memory of **Professor Helena Rašková, MD., DSc.***

Professor MUDr. Helena Rašková, DrSc., nestor of Czech and Slovak pharmacology and toxicology, would celebrate the 100th anniversary of her birth. She was born in 1913 in Laussane in a physician's family of a Czech father and Russian mother. The entire life of Professor Rašková was, however, bound to Slovakia. As she put it herself, one half of her heart belonged to the Czech lands, while the other half belonged to Slovakia.

As early as in 1946, she established contacts with Professor František Švec, Head of the Institute of Pharmacology at the School of Medicine, Comenius University in Bratislava, and since that time, until her last days, she had been in continuous touch with her Slovak colleagues, mostly pharmacologists and toxicologists. Prof. Rašková has significantly contributed to the establishment of pharmacology as an independent scientific discipline in the world context and established the Czecho-Slovak Pharmacological School of the 1950s and 1960s.

Due to her efforts, after agreement with the Czechoslovak Physiological Society in 1959, the Czechoslovak Society of Pharmacology was formed. She was repeatedly elected President of this society until 1970. Later she was Honorary President of the Czech Society for Experimental and Clinical Pharmacology and Toxicology and with her sustained vitality she remained in the center of many pharmacological as well as non-pharmacological activities. She used her authority in the foundation of a pharmacological laboratory within the Institute of Organic Chemistry and Biochemistry of the Czechoslovak Academy of Sciences (CzSASci). In 1963, she succeeded to make this laboratory independent and to form the Institute of Pharmacology with headquarters in Prague and with an affiliated branch in Bratislava, together with a farm for laboratory animals in Dobrá Voda, close to Bratislava. The Slovak part of this Institute of Pharmacology of the CzSASci was the basis of the present Institute of Experimental Pharmacology and Toxicology of the Slovak Academy of Sciences.

In 1961, together with Professor Uvnäs from Sweden, Professor Rašková established an independent international society of pharmacology, IUPHAR, of which she was an honorary member until her last days. In addition to the obtained goals in our experimental pharmacology of the 2nd half of the 20th century which are linked with the name of Prof. Helena Rašková, her initiative was of key importance also in our history of “Drug Toxicology” and “Clinical Pharmacology”. In 1963, the Czecho-Slovak toxicological section of the Pharmacological Society was among the founding members of the European Society for the Study of Drug Toxicity (predecessor of the present EUROTOX).

Following the split of Czechoslovakia in 1993, Prof. Rašková initiated the organization of periodical conferences in toxicology that have been organized alternatively in the Czech Republic and in Slovakia. Despite her advanced age, she actively participated in each of these conferences and, due to her immense professional credit, managed to ensure the participation of leading lecturers from abroad. This contributed greatly to improving the niveau of the conferences along with the interest of other countries in Slovak toxicology within its integration into the European Union.

The range of her scientific activities was voluminous and miscellaneous. She published more then 500 scientific papers and several books dealing with anesthetics, curare-like agents, anti-thyroids, analeptics, hypnotics, antimetabolites, drugs from plants, handbooks of pharmacology, etc. Remarkable international attention was given to her original complex of studies on pharmacology of bacterial toxins, on their effects, on non-specific resistance, as well as on the fate of drugs in the organism.

The wisdom of Professor Rašková, her generosity, kindness and hard work made it possible to create a positive environment for research and education, although many a time she had to fight hard to achieve her goals. All the pharmacologists and toxicologists in the Czech and Slovak Republic remember Prof. Helena Rašková, our teacher, scientific mother, grandmother and great-grand mother with special affection.

******

Several articles of this issue present original results on beneficial and protective effects of quercetine and its derivatives in various experimental models, such as *in vivo* intestinal inflammation, the activity of sarcoplasmic reticulum Ca^2+^-ATPase or inhibition of aldose reductase activity. Flavonoids are increasingly viewed as beneficial dietary components, considering their well-established antioxidant and antiradical properties. In addition, flavonoid compounds exert many biological effects, including enzyme inhibition (lipoxygenase, cyclooxygenase, nitric oxide synthase), immune cell modulation, etc. Quercetin (3,3’,4’,5,6-pentahydroxyflavone) (Q) is normally present in plants as a glycoside as quercitrin or rutin. Quercetin belongs to the most potent scavengers of reactive oxygen species Quercetin and other flavonoids dramatically reduced oxidative stress *in vitro* in lymphocytes. Moreover, quercetin and its derivatives were found to be inhibitors of the *Helicobacter pylori*-stimulated respiratory burst of neutrophils and noncompetitive urease inhibitors, indicating that they may be potentially useful in the therapy of other gastro-intestinal diseases. A series of synthetic derivatives of quercetin was prepared to improve antioxidant and other beneficial properties of the flavonoid. Since aldose reductase is involved in the pathogenetic way of inflammation, its blockade should ameliorate tissue injury induced by inflammatory processes. Quercetin derivatives were tested also for their inhibitory effect on aldose reductase.

**Michal Dubovický, PhD.**

Institute of Experimental Pharmacology & Toxicology

Slovak Academy of Sciences, Bratislava, Slovak Republic

E-MAIL: michal.dubovicky@savba.sk

